# Supplementary material for: Patterns of Intron Gain and Loss in Fungi
Source: PLoS Biol. 2004 Nov 30;2(12):e422. doi: 10.1371/journal.pbio.0020422 (PMC532390; doi:10.1371/journal.pbio.0020422)
Supplement: Table S1 — Also available at http://genes.mit.edu/NielsenEtAl/. (4.3 MB ZIP). [file pbio.0020422.st001.zip › NielsenEtAl/html/557.html]

AN9119.1.NCU05117.1.MG04717.1.FG02662.1


```
 CLUSTAL W (1.82) Multiple Sequence Alignments - Introns Inserted


Sequence 1: NCU05117.1	628 aa
Sequence 2: MG04717.1	614 aa
Sequence 3: FG02662.1	537 aa
Sequence 4: AN9119.1	587 aa
Alignment Length: 669 aa
Number Identitical Residues: 204 aa
Alignment Score (without introns) 11235


MG04717.1 	MTS~IADLFK0S----SGVPSKRKLDPVRDPSKQFDSLCLGFLTHIQW~TD~EIYKSAKL
NCU05117.1	-MN~VDELFQ0K----AGVPSKRKLDPIRDP-----------------~-N~EIYKSAKL
FG02662.1 	MAS~VDEIFK0S----SGISGKRKLDPIRDP----------------I1QD~EIYKSAKM
AN9119.1  	MTS0LCLSLT~KKKPLAAASAKRKLDITKDP-----------------~-N1ELYKAAKL
          	  . :   :  .... :. ..*****  :**                   : *:**:**:

MG04717.1 	NPSSS-RHAQVQDD----DDVEAGPAPPEDD--EDGDYG-PSAPPEDDIP--DDDEDGRF
NCU05117.1	SSNGS-RHAQVEDG----DDIEAGPAPPPEDGEDDGDYG-PSAPPVEDD---GDDEEGRF
FG02662.1 	SSSSSNRHAQVDDAQDEDEDMEAGPAPPPEE----EDFG-PELPP--------DDDEGRF
AN9119.1  	EANGD-----VKSK---------GKAPMVEDEEENDDYAGPELPPDFDAEDVADDEEGRF
          	.....     *..          * **  :: ... *:..*. **  .  .  **::***

MG04717.1 	FGGGITRQEAEVLDFVESADSAAPP~---ADDKFDAAWLRRTALALEKKINRNAELRARH
NCU05117.1	FGGGITAQEKEILDFMDNNTAAPAD~DLLTTDKIDLSWLKKTALNFEKRITRNAELRARH
FG02662.1 	FGGGITKQESEILDYVDEADAGNAP~-----EKIDAAWLRKTALNFEKRISKNAELRAKF
AN9119.1  	FGGGMERETAQAMDYIEEQDKGELT0N----EKYDVAWLRRFALNFEKKISKNAELRGKF
          	****:  :  : :*:::.   .    .    :* * :**:: ** :**:*.:*****.:.

MG04717.1 	ADDPAK~FIDSEADLDAAVRNLSILAEHPSLYPEFARLGIAGSLTGLLAHENTDIALAAV
NCU05117.1	EDDPSK~FIDSEADLDASIKALSILSEHPELYADFARLGCVSSLVSLLAHENTDIAIDAV
FG02662.1 	EDEPQK~FIGSEADLDADVKGLSLLSEHPELYPEFVKTGCVASLVSLLAHENTDIAIDAI
AN9119.1  	ENDPQK2FMASEADLDAEIKSLSILSEHTDLYPEFAKLGCVGSLVSLLSHENADIAIDAI
          	 ::* * *: ******* :: **:*:**..**.:*.: * ..**..**:***:***: *:

MG04717.1 	QIVAELTGDDVEGATEDDWAALVDSL~LEADLLSLLVSNLERLDEAGDEADREGVYRILE
NCU05117.1	EIINELTDEDVA-ASDEQFSALTDAL~LEADLLGLLVSNFSRLDEQ-QEADRTGVYHALS
FG02662.1 	EIMGELTDEDVS-AEDEQWNGLVDAM~MDADLLGLLVSNFSRLDED-DESDRSGIYHALG
AN9119.1  	QIISELTDEDVE-AEQEHWDSLVNAM0LDCDLIELLAQNLSRLNEE-EEADRAGVYYVLG
          	:*: ***.:**  * ::.: .*.::: ::.**: **..*:.**:*  :*:** *:*  * 

MG04717.1 	~VVENLCGGRPATAETVGRCDPLLKWLLARATKAEEP-VSQNKQYAAEMLAILAQASPVN
NCU05117.1	~IIENLCSRR-ETADQIGKHTELLEWLLSRAKKSESPTVSQNKQYAAEILAILVQSSPAN
FG02662.1 	~VIENLCSRQ-SVAERVGEDEKLLQWLLQRIQRKEDV-VSQNKQYAAEILAILSQMAVAN
AN9119.1  	1VFENLSSQV-HIAEKIGEDSNIMPWLLARIQKKERP-VSQNEQYSAEILAILLQSSTKT
          	 :.***..     *: :*.   :: *** *  : *   ****:**:**:**** * :  .

MG04717.1 	RDRLAR-DLDAVEPLLQLVAPYRARDPERGGDEEEFMENCFEALTCIADRPEGKQKFVDA
NCU05117.1	RRRLASDELNAVDTLLTLIAPYRRRDPERGSFEEEYMENLFECLTCLVDDPLGKTKFVEA
FG02662.1 	RTQLIR--LDAVDLLLQLAAPYRRRDPDKGGEEEEYMENIFASLTCLADEAAGKSKFIDA
AN9119.1  	RERFIS--LDGVDVLLQLLSQYRKRDPEKDSDEEEYAENLFDCLVCLVDDDAGKEKFLEG
          	* ::    *:.*: ** * : ** ***::.. ***: ** * .*.*:.*   ** **::.

MG04717.1 	EGYALCLVLIGSP--TAKKARTPALRLLDHAVAQSREAGVCEQVVEYGGLKTLFTLFMKS
NCU05117.1	EGVELCLLILSSADIKGKLSKPACLRLLDHAASFS--SEVCLKIVEAGGLKTLFTLFMSG
FG02662.1 	EGVELCLIMLKEG----KKSKPPSLRLLNHAAGGNSGVEVCQKIVEAGGLKTLFTLFMKT
AN9119.1  	EGIELVQIMLKEG----KFSKLRAIRTLDHALGGLEGVAACERLVEVAGLRTIFGMFMRK
          	**  *  ::: .     * ::  .:* *:** .      .* ::** .**:*:* :**  

MG04717.1 	~SSSAKASSKKR--------DPKVVEHLISIFACMLRLLPTDS----------------A
NCU05117.1	~DKSSDSTKKKNKGGKTPTLSKQDTEHLLGIFASMLRHLPATSSSPPSSSVTDDSETSGA
FG02662.1 	~Q------------------DHRLAEHLVEIFASMLRLLPANS----------------A
AN9119.1  	0Q------------------EHQIIEHLLGIFCSLLRLLPGGS----------------A
          	 .                  . :  ***: **..:** **  *                *

MG04717.1 	ERIRTLAKFVEKDYEKTERLVLLRREYAGRVAAVD-------AAGMD---ELDEDERFSR
NCU05117.1	ERIRLLAKFVEKNYAKTSKLIRLRREYAARVSQVDEAIKADAASSPE---QFDEDEAFSR
FG02662.1 	ERIRTLAKFVEKDYAKITKLIKFRRDYVARLSLAEQKNDAEKATTSADDRETAELEWLSR
AN9119.1  	PRIRTLAKFMEKDYEKTEKLVKLRRDYASRVSPVEQIIEKERKQFTEEEQKAMEAEWLSR
          	 *** ****:**:* *  :*: :**:*..*:: .:.  . .      .. :  * * :**

MG04717.1 	RLDAGLFVLQ~SIDVILAW~LVAEDDGAKRKIQTLLADRDETLELLSESLKE~QADAVDV
NCU05117.1	RLDAGLFCLQ~TIDVILAW~LIAEDTGCRAKIRELLKDRDEDFGVLARTLRE~QMAGVDS
FG02662.1 	RIDAGLCTLQ~TIDAVLAW2YSLS--GTKRYH--------------------~-------
AN9119.1  	RFDAGLFSLQ0LIDVILAW~LVAEDDGAKMKIVSILSDRDEDLSLIRSTLQE1QIEGLSE
          	*:****  **  **.:***    .. * :        . ..       :  . .  . . 

MG04717.1 	ETENGKEMHDMLTTLREFLQD
NCU05117.1	ETEEGRDTGEMLKALLEFLE-
FG02662.1 	---------------------
AN9119.1  	DEPGQKDHKEMLQTLLQFL--
          	.     .  .   :  .
```
